# A MEMS-Enabled Deployable Trace Chemical Sensor Based on Fast Gas-Chromatography and Quartz Enhanced Photoacousic Spectoscopy

**DOI:** 10.3390/s20010120

**Published:** 2019-12-24

**Authors:** Stefano Zampolli, Sandro Mengali, Nicola Liberatore, Ivan Elmi, Luca Masini, Michele Sanmartin, Roberto Viola

**Affiliations:** 1 Institute for Microelectronics and Microsystems, Italian National Research Council CNR-IMM, 40129 Bologna, Italy; zampolli@bo.imm.cnr.it (S.Z.);; 2 Consorzio CREO, 67100 L’Aquila, Italy

**Keywords:** MEMS GC, QEPAS, CBRN, forensic, safety and security

## Abstract

This paper reports on a portable selective chemical sensor for hazardous vapors at trace levels, which combines a two-stage purge and trap vapor pre-concentration system, a Micro-Electro-Mechanical-System (MEMS) based fast gas-chromatographic (FAST-GC) separation column and a miniaturized quartz-enhanced photoacoustic spectroscopy (QEPAS) detector. The integrated sensing system provides two-dimensional selectivity combining GC retention time and QEPAS spectral information, and was specifically designed to be rugged and suitable to be deployed on unmanned robotic ground vehicles. This is the first demonstration of a miniaturized QEPAS device used as spectroscopic detector downstream of a FAST-GC separation column, enabling real-world analyses in dirty environments with response time of a few minutes. The main modules of the GC/QEPAS sensor device will be described in detail together with the system integration, and successful test results will be reported and discussed.

## 1. Introduction

In the aftermath of industrial accidents or CBRN (Chemical, Biological, Radiological, Nuclear) events, there is a need for rugged and compact chemical sensors that, carried by hand or onboard unmanned ground vehicles and brought at short distance from suspicious items, are suited to detect traces of chemical threats by sniffing the air. A particularly challenging chemical sensing application is the selective detection of sub-ppm (part-per-million) concentrations of hazardous molecules (e.g., nerve and blister agents or toxic industrial chemicals) in a complex matrix of interfering species, as can be expected in the aftermath of a CBRN event. A sensor for such application scenario should combine high sensitivity with the ability to identify the threat even in the presence of fuels, solvents, detergents or any other interferent that is likely to be present on the scene. Furthermore, the sensor should be fast (few minutes measurement cycle time) and resilient to poisoning and saturation.

Most systems used nowadays by first responders in CBRN scenarios are small handheld systems which are easy to use and carry, give fast response at low concentrations, but generally lack the selectivity necessary to correctly identify threats without false positives [1,2]. As examples of best available technologies, the ChemPRO by Environics [3] couples ion mobility spectroscopy to solid state gas sensors. Other military grade IMS based sensors have excellent selectivity and quick response time, but are often limited to a specific set of chemicals [4] and generally prone to false positives in cases of some strong background matrices [5].

Nowadays, the most relevant characteristics of high sensitivity, high selectivity without false positives, portability and resilience to poisoning or saturation have been partially met only by a few portable gas-chromatography and mass spectroscopy (GC/MS) instruments [6,7]. The Hapsite ER GC/MS by Inficon [8] uses a Non Evaporable Getter (NEG) Pump to provide the vacuum necessary for the MS operation, making it the only actually portable GC/MS system, while the Griffin G510 by FLIR Technology [9] uses a turbo-pump for the MS vacuum, which prevents the system from being transportable once the pump is operating. As a matter of fact, the Griffin G510 device needs to be put on a stable surface before being switched on. Nevertheless, both of these GC/MS systems provide excellent identification capabilities through the combination of the selectivity achieved by GC separation and acquisition of mass spectra. Other portable GC systems exist, relying on photoionization detectors (PID) [10], flame ionization detectors (FID) [11] or thermal conductivity detectors (TCD) [12], but their identification capability relies only on the gas-chromatographic retention time. This level of selectivity is not sufficient for unambiguous detection of specific target molecules in a complex real-world matrix as expected in CBRN scenarios. As a matter of fact, the application addressed by our work is the detection and unambiguous identification of specific harmful molecules inside an unknown matrix of interfering gas species like gasoline vapors, explosion products or exhaust gases from trucks, tanks or industrial facilities. The complete qualification of a GC separation column to handle the identification of a single harmful compound inside such an unknown mixture is not feasible, and a second identification capability besides the GC retention time is necessary. Up to nowadays, the only partially fieldable option has been the GC/MS technique.

In the framework of the Horizon 2020 “ROCSAFE” project [13], several portable sensing systems are being designed to be deployed on robotic ground vehicles (RGV) and robotic air vehicles (RAV), with the aim of ensuring the safety of crime scene investigators by reducing the need for them to enter high-risk scenes when they have to determine the nature of threats and gather forensics. The GC/QEPAS sensor described in the next sections is the ROCSAFE high-end chemical sensor for detection and identification of trace amounts of nerve and blister agents and of some toxic industrial chemicals. To the best of our knowledge this is the first report of the successful hyphenation of a QEPAS detector with a GC column to achieve unambiguous identification of a chemical from inside a sample mix.

## 2. Sensor Description

**Figure 1:**
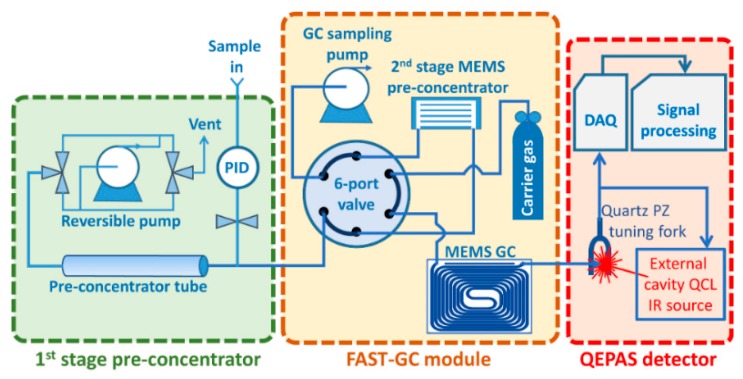
Sensing chain of the gas-chromatographic (GC)/quartz-enhanced photoacoustic spectroscopy (QEPAS) sensor.

The complete GC/QEPAS sensing chain is schematically shown in Figure 1 and consists of the following main functional units:

- a first-stage purge and trap pre-concentrator based on a commercial stainless-steel tube packed with a graphitized carbon sorbent;
- a FAST-GC module including a MEMS-based second-stage pre-concentrator [14] and a MEMS-based 5.3 m long FAST-GC separation column [15];
- a QEPAS sensor using a tunable external cavity quantum cascade laser IR source.

In order to reach ppb level sensitivity and achieve a high pre-concentration factor, it is mandatory to sample a large amount of air and release the trapped analytes in a possibly small volume. For security applications, the sampling time is critical and therefore the high volume must be sampled in a possibly short time. On the other hand, each sorbent material has a so-called breakthrough volume, which is the amount of sample volume allowed per gram of sorbent. To sample large volumes of air, large amounts of sorbents are necessary, which finally result in a relevant carrier gas volume necessary to flush the sorbent tube during desorption. Since a FAST-GC column requires a possibly sharp injection of a very small sample volume, a two-stage pre-concentration approach was chosen for this work: a first pre-concentration tube contains a relevant volume of sorbent, allowing for sampling a large air volume in a short time. During desorption this first stage pre-concentrator is flushed into a second-stage MEMS pre-concentrator, which acts as focusing injector into the GC column.

**Figure 2:**
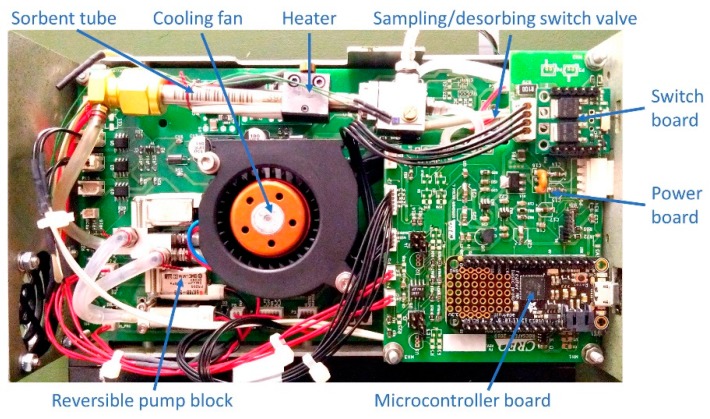
First stage sampling and pre-concentration module.

The first-stage sampling and pre-concentration module has been purposely developed for this work. It is the purge and trap device depicted in Figure 2, based on a commercial sorbent tube filled with Carbograph sorbent 2TD (Markes International), a miniature pump, compact manifolds and a set of micro valves enabling flow inversion between the sampling in injection step, together with the necessary heaters and a cooling fan. The first stage pre-concentrator allows to sample the air at a flowrate of 0.85 L/min and to desorb the preconcentrated vapors into the second stage pre-concentrator of the FAST-GC separation module. The sampling phase can last up to 5 min if necessary, allowing to pre-concentrate more than 4 L of air. In the context of the experiments reported in this paper, the first stage was used for the direct sampling of controlled air mixes from a 60-L glass box, or from gas distribution systems generating known concentrations of analytes. It is clear, however, that the first stage can easily be equipped with an external plug-in device for the thermal desorption of solid or liquid traces collected on a swab. Furthermore, it can be equipped with a commercial photoionization detector (PID) in-line with the sample input, as shown in Figure 1, which can monitor the overall concentration of the sample being acquired, with the aim of automatically self-limiting the sampling procedure once a sufficient amount of analytes is detected, and thus avoiding system poisoning or saturation.

**Figure 3:**
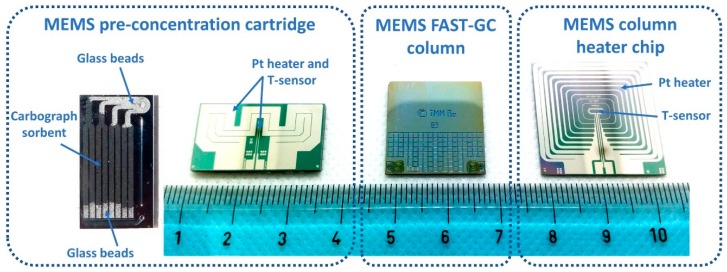
A packed Micro-Electro-Mechanical-System (MEMS) pre-concentrator (**left**) with backside integrated heater and temperature sensor, MEMS fast gas-chromatographic (FAST-GC) column (center, L = 5.3 m) and GC column heater chip (**right**). The scale unit is centimeters.

The MEMS second stage pre-concentration cartridge is shown on the left in Figure 3 and was fabricated on a 1.00 mm thick double-side-polished silicon wafer by Deep Reactive Ion Etching (D-RIE) of 8 parallel channels, each channel being 0.7 mm deep and 1.0 mm wide. The channels were then encapsulated by anodically bonding the silicon wafer with a 0.5 mm thick borofloat wafer with laser-machined in- and outlet holes. A platinum heater and a platinum temperature sensor were defined on the backside of the silicon wafer by means of a lift-off process. Further details on the fabrication procedure of the MEMS pre-concentrator are reported in [14]. Since very low volatility molecules, which are not efficiently desorbed, could be present in the sample mixture and saturate the pre-concentrator, a multi-bed sorbent mix was chosen: the main sorbent is a graphitized carbon black (Carbograph^®^ 2 mesh 80/100, specific surface 10 m^2^/g, LARA Srl, Formello, Italy). We then added a short shield area of glass beads (acid washed, mesh 70/100, Sigma Aldrich) at both sides of the pre-concentration cartridge, as highlighted on the left part of Figure 3. The desorption temperature of this second-stage pre-concentration device was set to 290 °C for all measurements reported in this work, and less than 4 s are necessary to heat the MEMS chip to said temperature.

The central part of Figure 3 shows the MEMS FAST-GC separation column, which integrates a double-spiral etched channel with 5.3 m length and a rectangular cross-section of 50 μm width by 80 μm height. In general terms, GC columns are conventionally defined as FAST-GC columns when their inner diameter is lower than or equal to 100 μm, yielding fast chromatograms (between 2 min and 10 min runtime) at very low carrier gas flow rates. In our case, the rectangular cross-section channel is fluidically equivalent to a circular channel with 70 μm inner diameter. The double-spiral shaped channel is fabricated on a silicon wafer by a D-RIE process. Silicon direct bonding with a second wafer featuring in- and outlet holes yields a buried channel, which was fluidically interconnected by means of two fused silica capillaries. The fabrication process of the MEMS column and its functionalization technique is reported in [15].

For this work, the FAST-GC column was internally coated by the company MEGA snc, Legnano (MI), Italy. The stationary phase selected for this application is a 0.3 μm thick 14% Cyanopropylphenyl, 86% Polydimethylsiloxane film. The heater and temperature sensor are not directly integrated on the MEMS column chip, since an additional silicon chip for temperature control, shown on the right part of Figure 3, was attached to the MEMS GC column. The FAST-GC column is operated at a constant pressure of 4 bar using nitrogen as carrier gas, resulting in a flowrate of 0.38 mL/min at the beginning of the GC temperature ramp, which starts at 50 °C and ends at 305 °C with a rate of 65 °C/min.

**Figure 4:**
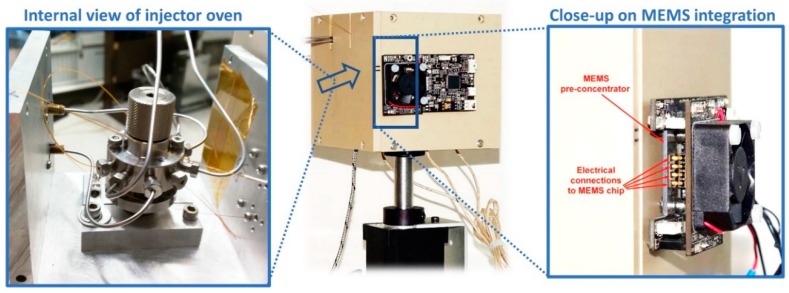
GC module integrating a 6-port valve inside an Aluminum oven with PEEK thermal insulation, with an internal view of the injector oven (**left**) and a close-up on the MEMS pre-concentrator and its temperature control electronics (**right**). The MEMS FAST-GC column is mounted on the other side of the oven (not visible).

To enable the analysis of high-boiling compounds and avoid sample condensation, the whole sensing chain is designed to be operated at high temperatures. The commercial 6-port injection valve (VICI-Valco) is integrated inside a custom designed small Aluminum oven which is thermally insulated by a polyether ether ketone (PEEK) housing (Figure 4), and polyimide tubes fitted with external stainless-steel metal braids (Microlumen Inc., Oldsmar FL, USA) are used as heaters for all tubes and transfer lines. The MEMS second-stage pre-concentrator and the MEMS FAST-GC column are mounted on specific supports, directly facing the thermal insulation of the injector oven, and the temperature control of the MEMS devices is provided by custom developed electronic PCBs interconnected to the MEMS heater pads with spring contacts for easy replacement of the MEMS chips.

**Figure 5:**
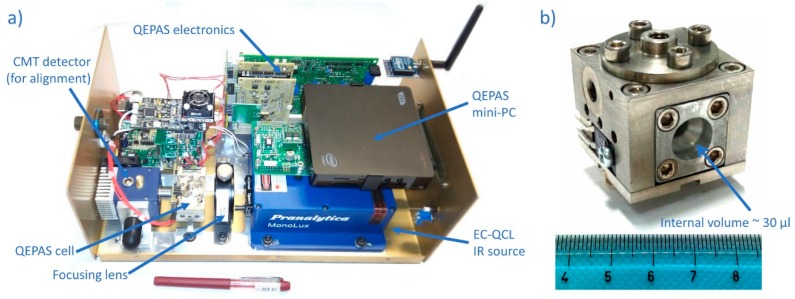
QEPAS module (**a**) and miniaturized QEPAS cell ((**b**), scale unit centimeter).

The QEPAS module, shown in Figure 5a, was completely designed and developed at CREO (Electro-Optical Research Centre, L’Aquila, Italy). It makes use of a quantum cascade laser source, (Monolux from Pranalytica, US), which continuously scans the thermal IR spectrum in the range of wavelengths between 8.8 µm and 9.9 µm, to perform the spectroscopic analysis of each of the substances released in a sequence by the FAST-GC module. As opposed to a mono-dimensional detector like FID or PID, which acquires a single signal at each detection interval during the chromatogram elution, the QEPAS sensor acquires an entire photoacoustic spectrum at each detection interval of less than 3 s, resulting in two-dimensional selectivity much like in GC/MS systems. Sensing is based on the piezoelectric transduction of the photo-acoustic signal that is generated when laser radiation is absorbed by the vapor and that reproduces the infrared spectral fingerprints of the substance. Signal transduction is accomplished by a standard commercial quartz crystal tuning fork resonating at 32,768 Hz. The amplitude of the laser radiation is modulated at the resonance frequency of the tuning fork, and is focused between the two prongs of the fork, where radiation is absorbed producing a photo-acoustic signal. The acoustic waves excite the prongs of the fork in opposing directions, thus generating a piezoelectric signal correlated with the intensity of the photo-acoustic excitation.

Signal intensity is proportional to laser intensity, to the absorption cross section of the substance in the QEPAS cell, and to its concentration. Moreover, an acoustic micro-resonator is implemented to amplify the photoacoustic excitation. A detailed explanation of the QEPAS technique and its application to gas sensing is reported in [16]. High sensitivity, down to part-per-trillion level, can be achieved [17]. QEPAS spectra allow the same identification capability obtained by classical IR absorption spectroscopy (IRAS). QEPAS based detection of broadband absorbing molecules using a widely tunable QCL is reported in [18]. The QEPAS sensor, however, is much more compact, robust and cheap than an analogous IRAS sensor of similar sensitivity, which is based on multi-pass cells and cooled cadmium-mercury-telluride (CMT) detectors. Moreover, the QEPAS technique enables the miniaturization of the interrogation cell [19], and is therefore particularly suitable for coupling with FAST-GC (which is itself characterized by miniaturized volumes). For our sensor we developed a novel miniaturized interrogation cell sized 30 mm × 30 mm × 25 mm, as shown in Figure 5b. The main innovation introduced respect to existing QEPAS cell relies on the miniaturization of the internal volume for analysis, that is of ca. 30 µL. This allows the hyphenation of our QEPAS detector to the FAST-GC column, avoiding dilution or remixing of peaks inside the cell. The cell includes the quartz tuning fork, a custom developed micro-resonator consisting of two small stainless-steel tubes of 0.9 mm internal diameter cut to 4.6 mm length, and two anti-reflective coated germanium windows (Edmund Scientific). Furthermore, the interrogation cell is made of stainless steel and is heated at up to 140 °C, this enabling the analysis of even low-volatility, sticky or chemically reactive substances.

The electronics, including a pre-amplifier board for the read out of the tuning fork signal and all the thermal controls, has been purposely developed. A commercial lock-in amplifier board (Femto LIA-BVD-150-H) and a mini PC unit have been also implemented. The custom developed software of the QEPAS sensor compares each acquired QEPAS spectrum with spectra from an internal database by means of a correlation analysis. We have used the Pearson correlation coefficient between the measured spectra and the spectra in our reference database. We will express this correlation coefficient as a percentage (e.g., a correlation of 95% corresponds to a calculated Pearson correlation coefficient of 0.95). The database spectrum with the highest correlation is cross-checked with the retention time of the GC separation. Only if the correlation exceeds 80% and the retention time is within the expected time window, a positive detection is reported. Using this algorithm, we obtained no false negatives, while all true positives were correctly identified.

**Figure 6:**
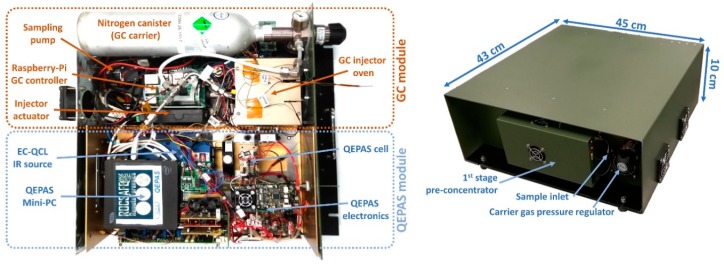
The complete GC/QEPAS sensor, open (**left**) and packaged (**right**).

The system is assembled as shown in Figure 6 left, including a 1-L canister with nitrogen carrier gas pressurized at 40 atm enabling several days of continuous operation, and integrated into a rugged portable case (right) suitable for integration into the ROCSAFE robotic ground vehicle. The QEPAS module inside the assembled system is the same as reported in Figure 5a), without the CMT detector which was removed after laser alignment, and without the Zigbee module which was used only during preliminary testing of the QEPAS module.

The sensor has a peak power consumption of 200 W in the warmup phase, which lasts for about 15 min. After warmup, the average power consumption during analysis is of 150 W. It completes one measurement cycle (from sampling to response, including the time to complete elution) in about 7 min, resulting in 17.5 Wh per cycle, and can be powered with a battery pack at 24 V. Most of the power is needed to keep the system at high working temperature in order to deal with high boiling compounds.

## 3. Results and Discussion

The GC/QEPAS sensor was tested in laboratory conditions with ppm and sub-ppm amounts of nerve agent simulants (dimethyl methyl phosphonate DMMP and dipropylene glycol methyl ether DPGME), a blister agent simulant (methyl salicylate) and a real blister agent sample (sulfur mustard HD). Some of the substances above were tested together with ethanol, propanol, gasoline and paint fumes at much higher concentrations, to simulate the impact of interferents on the detection and identification of traces in a real scenario. The details of the most significant experiments together with the total absorption chromatograms and the photoacoustic absorption spectra will be reported below. These results demonstrate, for the first time, the capability of a GC/QEPAS sensor to retrieve the IR spectral fingerprints of different compounds of a mix, even if at trace level and in presence of strong interferents.

### 3.1. Simulants of Nerve Agents: DMMP and DPGME

The preliminary sensing tests were performed at CREO, using a hotplate to evaporate a known volume of liquid samples inside a chamber of known volume (60 L). An upper limit of the resulting concentration can be estimated if we ideally assume the total vaporization of the injected liquid. But, depending on the different volatility of the tested compounds and considering partial condensation on the cold inner walls of the gas box, the real vapor concentrations are expected to be significantly lower, and the reported concentrations are therefore to be considered upper limits.

**Figure 7:**
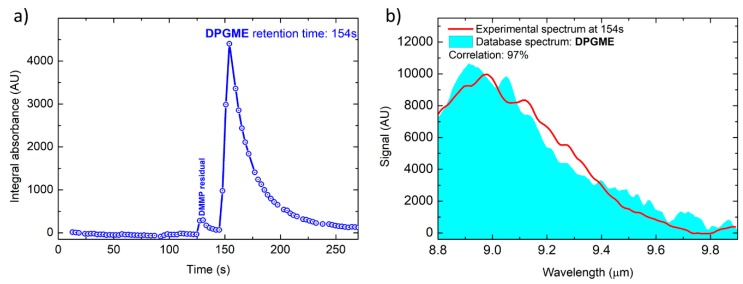
Total absorption chromatogram (**a**) and DPGME spectrum acquired at T = 154 s compared with the library spectrum (**b**).

The first result, shown in Figure 7, refers to the evaporation of 1 microliter of liquid DPGME, which results in a vapor concentration below 3 ppm. The content of the chamber was sampled by the first stage pre-concentrator at a flowrate of 850 mL/min for 60 s, resulting in a sample volume of 0.85 L. The chromatographic peak of DPGME (Figure 7a) and the corresponding QEPAS spectrum (Figure 7b) are clearly detected, allowing to identify DPGME with a correlation of 97%. It is worth to note that also a small peak corresponding to residual DMMP from previous tests was detected, even though the gas box is opened and vented before each test.

Please note that the total absorbance chromatogram resembles a PID chromatogram, since it plots the variation over time of what we named ‘Integral absorbance’, which is calculated as the mean value of the QEPAS spectrum acquired at each point of the chromatogram, and thus is proportional to the concentration of any IR absorbing analyte passing across the QEPAS cell. The plotted integral absorbance is the mean value of the measured QEPAS spectra. Each spectrum is calculated by subtraction of the reference background signal, which is acquired just before the injection into the GC, to the signal currently acquired. If only carrier gas travels through the QEPAS cell, the current signal and the background signal remain almost the same, except for offset variation due to thermal fluctuations and slow thermal drift that can induce a positive or a negative shift in the computed Integral Absorbance.

Anyway, either by using a PID, or by measuring the Integral absorbance, as in our case, only the intensity of the chromatographic peaks eluted from a GC column can be obtained together with their retention time. Since peaks can have similar retention times, especially in case of real-world sample mixtures, this doesn’t allow unambiguous identification of the target compounds. It is noteworthy that the typical application of this sensor is not the detection and identification of mixture of target analytes, but it is the unambiguous identification of a single target analyte in a complex real-world mixture, where hundreds of unknown interfering gas species may be present at relevant concentrations. However, thanks to the implementation of the QEPAS detector, we do also acquire the IR absorption spectrum of the sample for each point of the acquired chromatogram. Identification is obtained from the spectrum; the retention time is just a cross-check to eliminate false positives. The GC/QEPAS combination significantly improves the ability to identify substances with no false positives respect to a GC/PID, especially for a fast chromatogram like the ones reported, whose peak separation capability is intrinsically limited by the total elution time of only 2–3 min. In particular, the QEPAS detector can completely eliminate the ambiguity that would derive from using a PID, whenever the QEPAS spectrum measured is clearly attributable to a certain substance. That is, when substances with similar elution times are not present simultaneously in the analyzed mixture, or at least they are not present in equally relevant concentrations, as typically occurs for the most dangerous substances in the case of accidents or chemical attacks. Therefore, the spectral information acquired by the GC/QEPAS sensor can be compared to GC/MS spectra sequences rather than to GC/PID chromatograms

The availability of QEPAS spectra significantly overcomes the identification limits deriving from the use of the fast chromatogram alone, both because the latter cannot separate many peaks in the short elution time available to be ‘fast’, and also because the spectrum allows to unambiguously identify substances of similar or even equal retention times.

The broadening of peaks compared to conventional chromatograms can be explained by several factors: (1) even though sampled chemicals are concentrated into the MEMS cartridge to improve sensitivity and allow a fast injection, tens of seconds can be necessary to transfer the sample trapped from the MEMS pre-concentration cartridge into the column. This is due to the low carrier flowrate used to flush the MEMS cartridge during the split-less injection into the GC, and also to the fact that most of the tested compounds are high boiling and sticky molecules. An optimized MEMS cartridge is being designed, aiming at reducing the time needed for injection; (2) the fast GC column should manage small quantities of analytes to give narrow peaks, whilst in our case the pre-concentrated sample can easily overcharge the column; (3) even though the miniaturized internal volume of the cell allows to follow the profile of the peaks eluted by the column avoiding coelution inside the cell, the actual working temperature (140 °C) may not be sufficient to completely avoid partial condensing of high boiling compounds, which translates into longer persistence inside the cell and in broader peaks.

To establish the sensitivity of the GC/QEPAS sensor, quantitative tests were performed in the framework of the ROCSAFE work package on sensor validation. It is important to point out that it is very complicated to generate precise concentrations of low volatility compounds like the targets used for this work, since the partial condensation of the vapors on some of the tubes and gas lines cannot be avoided, despite the efforts of heating all parts of the characterization system. Therefore, samples were generated by flushing a known nitrogen flow through the headspace of a cooled liquid sample and roughly estimating the expected concentration. Afterwards the exact concentration was quantified by sampling a 500 mL/min flow for 5 min into a Tenax tube and successively analyzing the Tenax tube by a calibrated thermal desorption GC/MS system. Because of this complex procedure, determining the limit of detection (LoD) for several compounds is beyond the scope of this work, and we will present the lowest concentrations which were successfully generated and detected, which are in any case relevant for the application scenarios but may be higher than the actual LoD.

**Figure 8:**
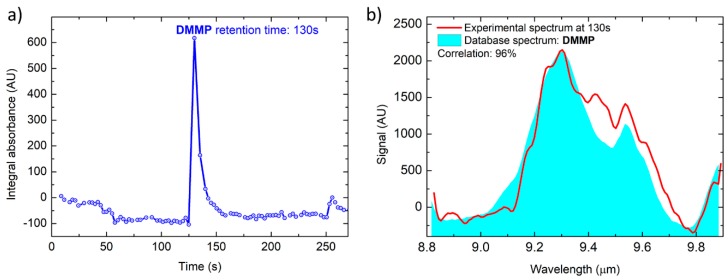
Total absorption chromatogram (**a**) and DMME spectrum acquired at T = 130 s compared with the library spectrum (**b**).

Figure 8 shows the results of a DMMP concentration, determined by GC/MS to be at 36 ppb (part-per-billion), sampled by the first stage pre-concentrator at a flowrate of 850 mL/min for 200 s, resulting in a total sample volume of 2.83 L. Even at this trace level concentration, the total absorption chromatographic peak of DMMP is clearly detected (Figure 8a) and the corresponding spectrum (Figure 8b) is identified with a correlation of 96% to the reference spectrum from the database.

### 3.2. Simulant of Blister Agents: Methyl Salicylate

**Figure 9:**
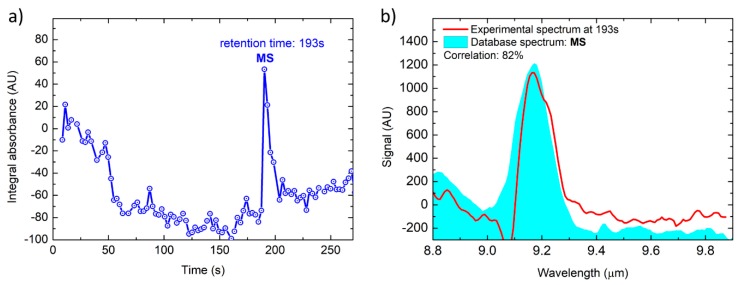
Total absorption chromatogram (**a**) and MS spectrum acquired at T = 193 s compared with the library spectrum (**b**).

The same quantitative test as performed on DMMP was repeated on methyl salicylate (MS). The quantitative GC/MS analysis determined the MS sample concentration to be 120 ppb, which was sampled by the first stage pre-concentrator at a flowrate of 850 mL/min for 300 s, resulting in a total sample volume of 4.25 L. Results are reported in Figure 9, showing that methyl salicylate is clearly detected and identified at this trace level concentration with a spectral correlation of 82%.

The peak at 193 s in Figure 9a allows us to identify methyl salicylate by means of the corresponding measured spectrum, which is shown in Figure 9b, even at this low concentration, which seems to be close to the limit of detection. In fact, the noise in the plot of Figure 9a is enhanced and more evident with respect to the other plots of Integral absorbance reported in this article. In particular if compared to Figure 7a, which show measurements corresponding to other tested compounds at much higher concentrations.

### 3.3. Real Blister Agent Sample: Sulfur Mustard HD (Yperite)

**Figure 10:**
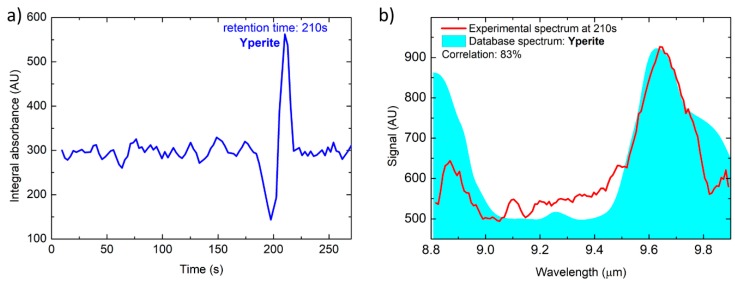
Total absorption chromatogram (**a**) and sulfur mustard HD spectrum acquired at T = 210 s (**b**).

A test on a real blister agent sample, namely sulfur mustard HD also known as Yperite, was successfully performed. In a specifically equipped laboratory, the headspace of a liquid Yperite sample kept at a temperature of 14.0 °C was flushed with a flow of 60 mL/min nitrogen, successively diluted in 1200 mL/min nitrogen, resulting in a calibrated Yperite concentration of 2.1 ppm. For safety reasons the GC/QEPAS sensor was operated wirelessly from another lab, and configured to sample at a flowrate of 850 mL/min for 300 s. The results are reported in Figure 10, showing that such trace concentrations of Yperite can be identified with a correlation of 83%. Please note that, in this case, the reference is an Infrared absorption spectrum from a FTIR library, since a reference QEPAS spectrum for Yperite was unavailable. This can explain the lower correlation value respect to the other tested and reported compounds. Furthermore, it is worthwhile to highlight that Yperite has weak IR absorption bands in the range of the IR laser source used in this work, which finally results in the Yperite sensitivity being two orders of magnitude lower than for DMMP and DPGME. In the test with Yperite, the background signal was acquired before the sampling phase of 300 s, so that an offset in the background signal was present before the first acquired spectrum. This explains the starting value of the plotted Integral absorbance of 300 (instead of 0) in Figure 10a.

### 3.4. Mix of Nerve Agent Simulants and Propanol

**Figure 11:**
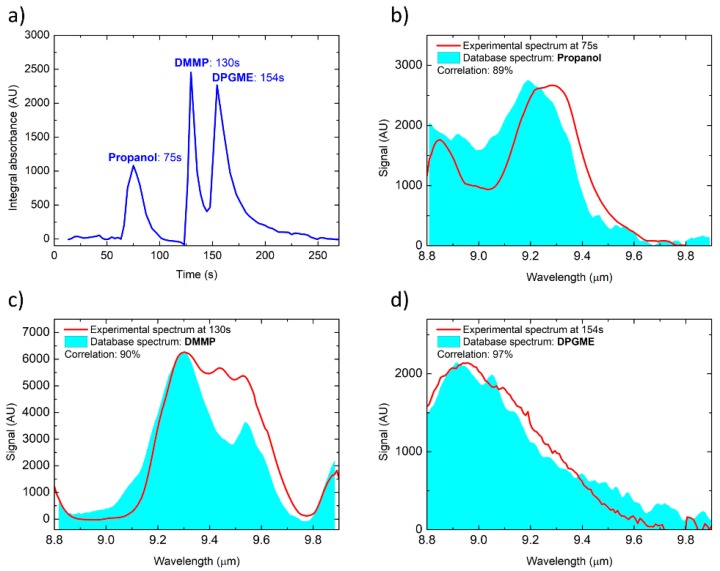
Total absorption chromatogram (**a**) of a 3-component mixture, and the correctly identified spectra of Propanol, DMMP and dipropylene glycol methyl ether (DPGME) at the respective retention times (**b**–**d**).

To highlight the sensor discrimination capability as enabled by the two-dimensional selectivity, a mixture of DMMP and DPGME in a strong background of propanol (isopropyl alcohol) was used for a further test cycle. All three of the compounds were successively sampled as headspace from three small vials for few seconds only, with a total pre-concentration time of 1 min. The exact amount of the three substances is therefore not known, but these tests mainly aim at demonstrating selectivity and identification capability, while the analysis of the quantification capabilities of the GC/QEPAS sensor are beyond the scope of this work. Figure 11a shows the total absorption chromatogram with the three peaks of the substances, while Figure 11b–d shows the three spectra as correctly identified by the QEPAS software. However, some mismatch appears in Figure 11c, which can be explained by the partial saturation of the signal due to the higher concentration of DMMP sampled from the mix, compared to the test reported in Figure 8.

**Figure 12:**
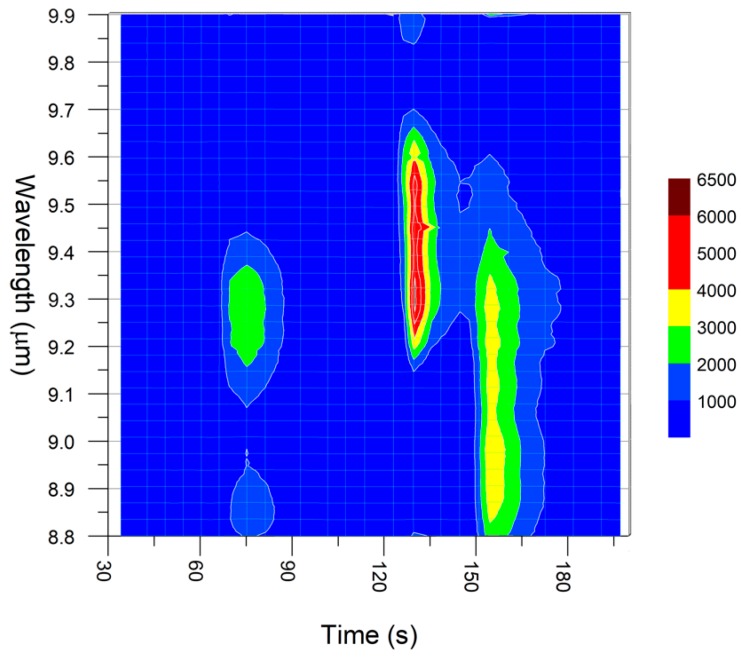
Color plot of all spectra acquired for the mixture of propanol, DMMP and DPGME.

A different visualization of the measurement done on the propanol mixture with DMMP and DPGME is shown in Figure 12, where the entire chromatogram of QEPAS spectra is plotted in a bidimensional color-plot, with the GC retention time on the X-axis and the QEPAS absorption spectrum on the Y-axis. This kind of plot clearly emphasizes the bidimensional selectivity achieved by combining the temporal gas-chromatographic separation with the chemical spectroscopic information of the QEPAS scans.

### 3.5. Nerve Agent Simulant DMMP with Gasoline Background

To demonstrate the capability of detecting and identifying nerve agent simulants also in presence of a strong background, as can be expected in a real-world CBRN scenario, a test using the 60-L chamber containing traces of DMMP (less than 1 microliter was evaporated) together with a beaker with liquid gasoline was performed. The chamber was allowed to reach a saturated gasoline vapor equilibrium before sampling into the first stage pre-concentrator of the GC/QEPAS sensor. A pre-heating of the first stage pre-concentrator, flushing high volatility molecules into exhaust, enables to significantly lower the gasoline background, and such method was configured in the sensor software for this test. Furthermore, less volatile gasoline constituents have no significant absorption in the wavelength range of the IR laser source.

**Figure 13:**
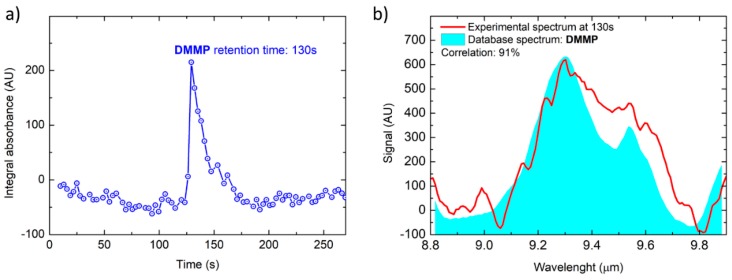
DMMP with strong gasoline background: total absorption chromatogram (**a**) and DMMP spectrum acquired at T = 130 s (**b**).

As can be disclosed in Figure 13, the sensor allows for the identification of a DMMP trace concentration (estimated below 0.1 ppm), despite the saturated gasoline background in the sample, which was at several orders of magnitude higher concentrations.

## 4. Conclusions

Hyphenation of a FAST-GC module for chemical separation with a QEPAS detector for chemical identification has been demonstrated for the first time. This was achieved by matching the miniature volumes of the core components of the FAST-GC, micromachined on silicon chip, with the internal volume of analysis of the QEPAS cell. It is worthy to note that the core components of the FAST-GC separation module (a MEMS pre-concentration cartridge and a MEMS column chip) and the miniaturized QEPAS analysis cell have been specifically designed and developed to allow such hyphenation. To reach the necessary sensitivity, a high-volume two-stage pre-concentration system was implemented, where the second stage MEMS pre-concentrator acts also as GC focusing injector.

The integrated GC/QEPAS sensor is a rugged and field deployable prototype for CBRN security applications and has been successfully utilized for the identification of simulants of hazardous chemicals, even in presence of high concentrations of common interferents, like gasoline and paint fumes. A typical analysis cycle lasts less than 7 min, including pre-concentration and GC column ramp. The entire system weights less than 10 kg, including the carrier gas source and the complete signal acquisition and processing hardware. Compared to portable GC/MS instruments today available on the market, the GC/QEPAS sensor achieves comparable sensitivity and identification capability, and also promises advantages in terms of portability (smaller and lighter), warm up time, sensing and cycle time, and costs.

Future work will include the optimization of the MEMS pre-concentrator to implement sharper injections, the increase of the QEPAS cell operating temperature and the further reduction of its internal volume, expecting enhanced chromatographic performance. Furthermore, we will pursue a further reduction of the sensor power budgets by introducing a new compact-GC technology patented by CNR-IMM, and a further miniaturization of the QEPAS cell to improve portability of the sensor towards handheld operation or onboard unmanned aerial vehicles (UAV).

## 5. Patents

A patent on the GC/QEPAS technology was filed in June 2019.
